# Case Report: Holistic dental care for a child with Hunter syndrome: Addressing dental ramifications, overcoming challenges, and enhancing quality of life

**DOI:** 10.12688/f1000research.146468.1

**Published:** 2024-04-15

**Authors:** Swagata Saha, Krishna Priya, Kavita Rai, Manju R, Krithika Shetty, Amitha M Hegde, Ananya Rao K, Dhvani Abhijit Tanna, Mohanaram S, Shreya S

**Affiliations:** 1 Department of Paediatric and Preventive Dentistry, A B Shetty Memorial Institute of Dental Sciences, Deralakatte, Mangaluru, Karnataka, 575018, India

**Keywords:** Hunter Syndrome; Dental Rehabilitation; General Anaesthesia; Multidisciplinary Approach; Oral Intubation; Dental Pain; Dental caries

## Abstract

Hunter syndrome (MPS II), an X-linked recessive lysosomal storage disorder, is a result of deficiency of the iduronate 2-sulfatase enzyme (IDS), leading to cognitive impairment, systemic organ involvement, and increased dental problems. This case report describes the management of a child with Hunter syndrome who was referred to the Department of Paediatric and Preventive Dentistry for pain in the upper front teeth. Intraoral examination revealed severe early childhood caries, prompting planning for full-mouth rehabilitation under general anaesthesia due to the child’s uncooperative behaviour.

In response to recommendations from the Department of Otolaryngology and the Department of Paediatric Surgery, a comprehensive treatment plan consolidated full-mouth rehabilitation in addition to adenoidectomy and inguinal and umbilical herniotomy procedures during a single session of general anaesthesia. Successful interventions were reflected in the uneventful one-month follow-up of the patient, highlighting the efficacy of the interdisciplinary approach.

The key takeaway underscores the importance of collaborative interventions, emphasising singular intubation for patients requiring recurrent hospitalisations, providing both monetary relief and reducing post operative healing time. Designed to address global developmental delay in the child, a personalised home care plan was also implemented. Evaluation of plaque and gingival indices before and after the home care regimen demonstrated a notable improvement, indicating an enhanced oral quality of life.

## Introduction

Hunter syndrome, an X-linked recessive mucopolysaccharide disorder, is characterised by a deficiency in the enzyme iduronate sulfatase, resulting in the accumulation of dermatan and heparan sulphates in various tissues. This disorder, which exhibits both mild and severe forms, is distinguished as an X-linked recessive condition from other autosomal recessive mucopolysaccharide disorders. The clinical manifestations of Hunter syndrome include macrocephaly, developmental delay, dysmorphic facies, skeletal abnormalities, joint contractures, hepatosplenomegaly, cardiac valvular disease, hirsutism, hyperkinesis, and rough behaviour. Inguinal hernias are reported in 60% of male patients, highlighting a characteristic feature of this syndrome. In addition, people with Hunter syndrome often experience umbilical hernias. Frequent otitis media and hearing problems are common secondary systemic manifestations, further illustrating the extensive impact of this disorder on various aspects of health. ^1^

The oral manifestations are prominent and involve a shortened and broad mandible, radiolucent jaw lesions, flattened temporomandibular joints, macroglossia, peg-shaped teeth with wide spacing, highly arched palate with flattened alveolar ridges, and hyperplastic gingiva. Charles Hunter’s early observations of this disease in children, dating to 1915, highlighted features such as a slowed learning process, tonsil and adenoid issues, severe respiratory infections, and numerous physical abnormalities, often increasing with age. ^2^

Hunter syndrome is extremely rare, affecting less than 20 cases per million births, and stands out as the rarest form of mucopolysaccharide disorder. It is important to note that the disorder can appear in successive births even without a family history of mucopolysaccharide disorders, making genetic counseling essential. ^3^

Children with Hunter syndrome often experience severe dental problems, including high dental caries, due to various factors such as malformed teeth, poor oral hygiene, and limited access to dental care. This case report illustrates the challenging but essential task of full-mouth rehabilitation for a child with Hunter syndrome, addressing these dental issues to improve their oral health and overall quality of life.

## Case report

**Figure 1.**
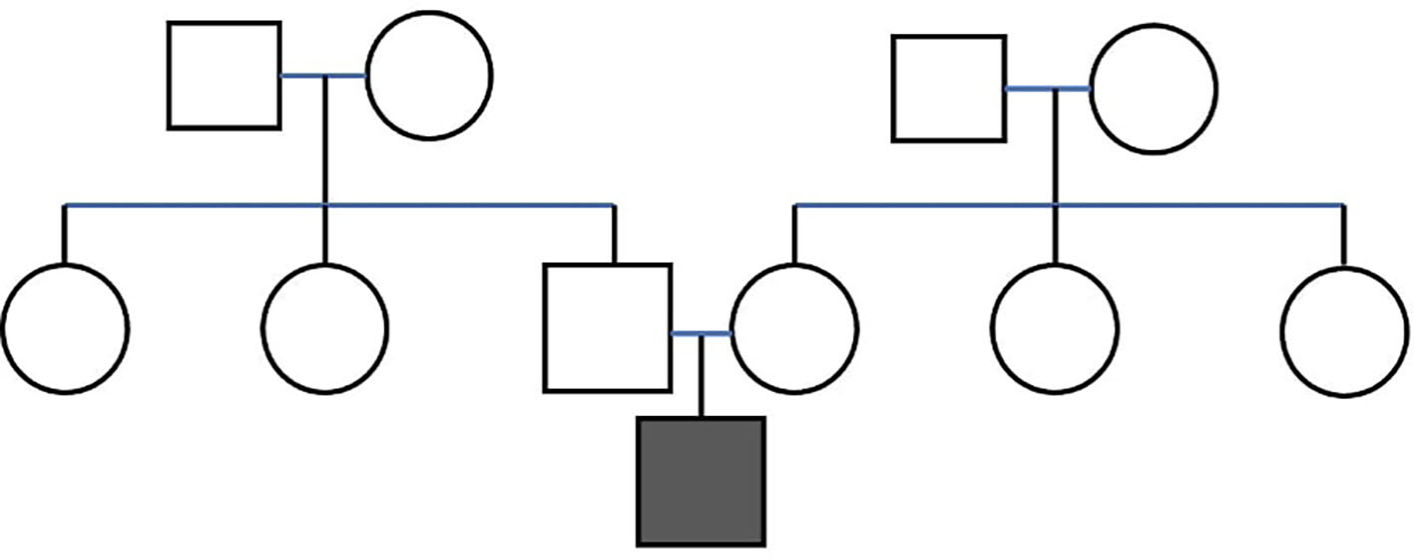
Pedigree chart of the patient.

A 4-year-old male patient presented to the Department of Paediatric and Preventive Dentistry at the AB Shetty Memorial Institute of Dental Sciences, Deralakatte, Mangaluru, with the chief complaint of decay in the region of the upper front tooth for the past month. The referral originated from the Department of Pediatrics at Justice KS Hegde Charitable Hospital, with a thorough family history analysis that revealed no hereditary patterns of the disease and parents involved in a non-consanguineous marriage ( Figure 1).

**Figure 2.**
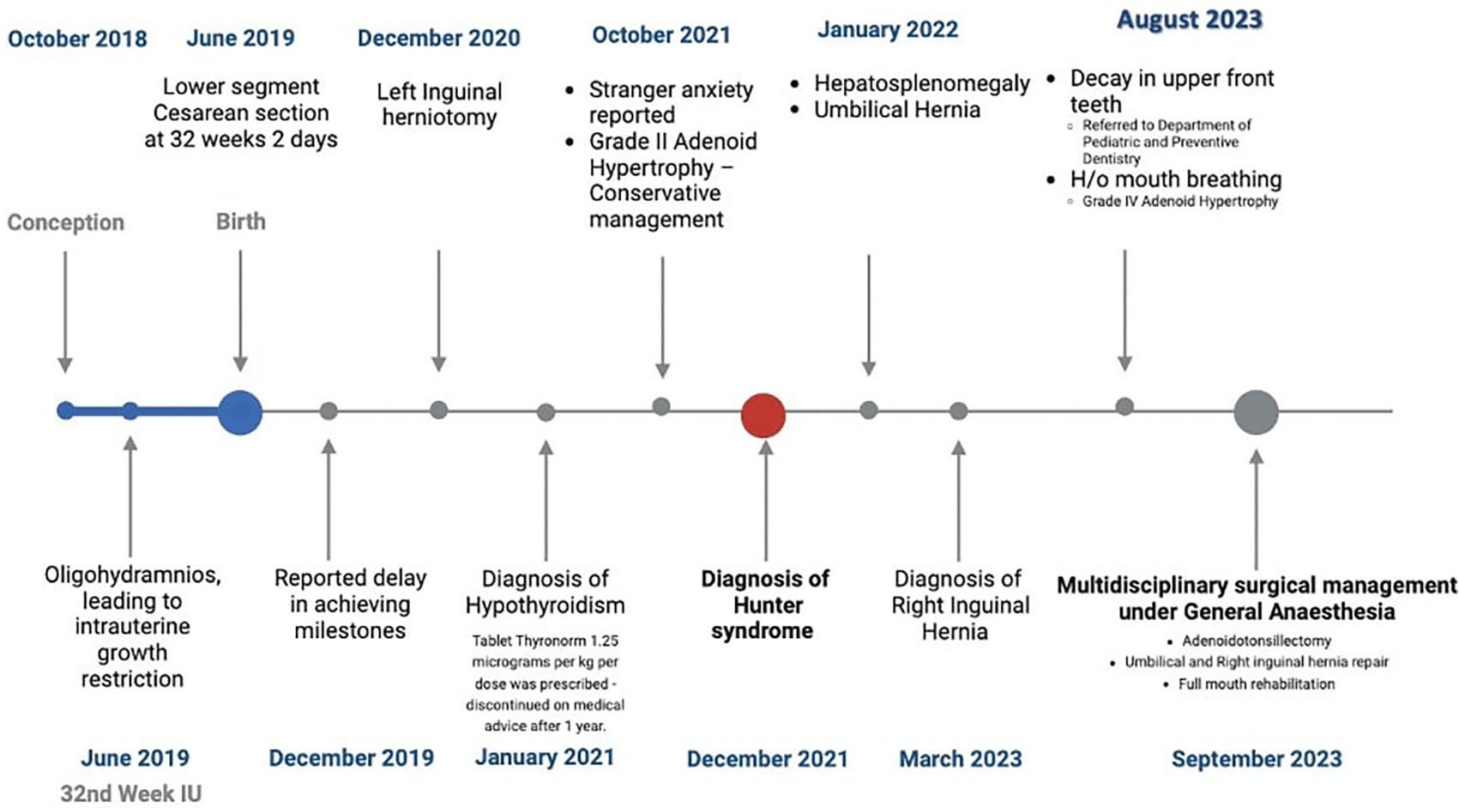
Timeline depicting the medical history of the patient.

During the 32nd week of intrauterine development, ultrasound revealed oligohydramnios, resulting in intrauterine growth restriction (IUGR). The subsequent timeline showing the major events is shown in Figure 2. The patient was diagnosed with Hunter syndrome through biochemical genetic testing at the Centre for DNA Fingerprinting at Kasturba Medical College, Mangalore. The assay using 4-methylumbelliferone confirmed the deficient activity of Iduronate 2-sulfatase (4.95 nmol/4 h/ml vs control), while the enzyme assay of Arylsulfatase indicated normal results. The diagnosis was confirmed by whole genome sequencing.

**Figure 3.**
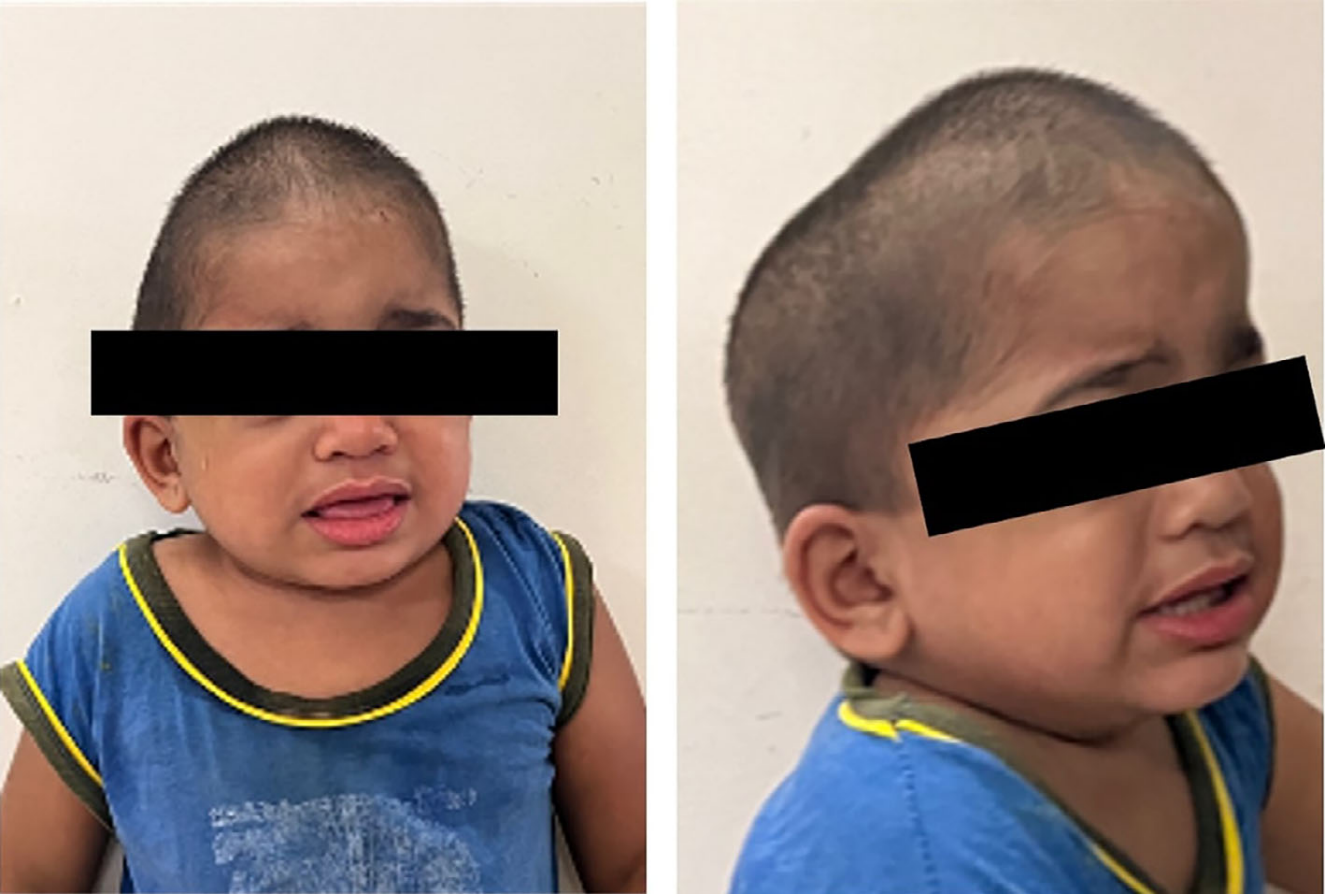
Extraoral photographs of the patient.

**Figure 4.**
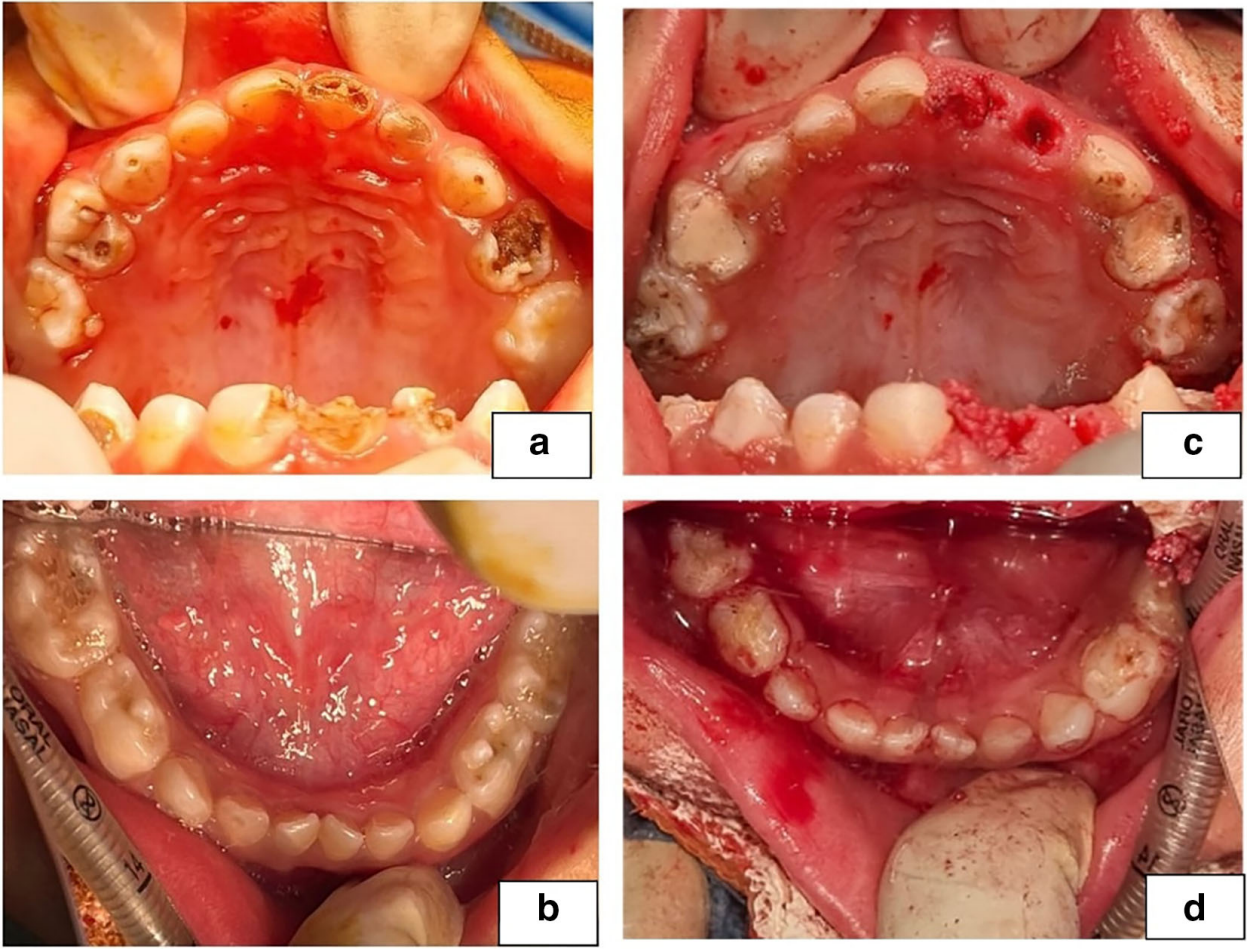
Pre operative (a – Maxilla, b - Mandible) and Post operative photographs (c – Maxilla, d - Mandible) of the patient.

After a year, the patient presented with complaints of pain in the upper front teeth, mouth breathing, and swelling over the right inguinal hernia (measuring 2 × 2 cm over the umbilicus and the right inguinal region, spontaneously reducible, and expanding on the cough impulse). On physical examination, the child presented with a clumsy gait, thick coarse hair and skin, generalised facial puffiness ( Figure 3) and brittle unkempt nails. On dental examination, severe early childhood caries was identified. Despite initial attempts to guide behaviour in the dental operating room, the child, who exhibited a Frankel rating of (--), displayed high apprehension and global developmental delay. Subsequently, a collaborative approach involving multiple departments was implemented to provide treatment under general anaesthesia. The Department of Otolaryngology performed endoscopic adenoidectomy to address grade IV adenoid hypertrophy, while the Department of Paediatric Surgery performed a umbilical and right inguinal herniotomy. Oral rehabilitation (Oral prophylaxis; Pulpectomy (Metapex) with respect to FDI Tooth Number 63, 64, Glass Ionomer Cement Type IX restoration with respect to FDI Tooth Number 65, 75, 55; Indirect Pulp therapy with respect to FDI Tooth Number 51, 53, 54, 74, 84, 85 (Biodentine); Composite Restoration with respect to FDI Tooth Number 63, 51; Extraction with respect to FDI Tooth Number 61, 62; Topical Fluoride application (APF gel)), was undertaken by the Department of Pediatric and Preventive Dentistry ( Figure 4). This comprehensive interdisciplinary effort aimed to address the diverse health concerns of the child efficiently and with due consideration of their overall well-being. After the procedure, the child received a comprehensive medication regimen and was reviewed after one week. One month after the procedure, the patient reported an uneventful follow-up, with no new dental complaints.

A holistic approach to subsequent oral care was diligently implemented. The personalised home oral care routine introduced a wide range of activities, including gum massage, blowing exercises, and oral motor drills, designed not only to stimulate oral sensory muscles but also to prevent potentially harmful coping mechanisms that arise from regression in daily activities. ^4^ Recognising the delicate nature of the oral epithelium, the integration of topical vitamin E into twice daily oral massage was intended to promote epithelization and overall oral health. ^5^ Confronting the challenge posed by the child’s inability to spit, a suite of recommendations was proposed, including the use of fluoride-free toothpaste that was safe to consume in minimal amounts, soft bristled or electric toothbrushes, routine tongue scraping, and post-meal mouth rinse facilitated by an irrigation syringe. Dietary modification, a critical aspect of this holistic care plan, advocates limiting the consumption of sweet and sticky foods ^6^^,^^7^ and incorporating probiotics. ^8^^,^^9^ The importance of regular mealtimes and family meals ^10^^,^^11^ further enriched the comprehensive care approach. During recall visits, the application of topical 5% sodium fluoride with casein phosphopeptide – amorphous calcium phosphate complexes (CCP-ACP) remineralisation paste was recommended every three to six months. This strategic intervention aimed to improve oral health over time. Assessment of plaque and gingival indices before and after implementation of the home care regimen revealed a significant improvement after 6 months of follow-up, underscoring improved oral quality of life.

## Discussion

This case report underscores the effectiveness of employing a multidisciplinary approach to address the various challenges encountered during a singular intubation procedure performed under general anaesthesia for a child. The decision to adopt this approach was rooted in the goal of mitigating possible side effects related to multiple intubations, a consideration that became important given the intricacies of managing a difficult airway. This approach not only led to a shorter hospital stay, thus reducing the risk of nosocomial infections and promoting cost effectiveness, but also proved to be notably advantageous for the child, especially considering that individuals with Hunter syndrome often require frequent hospital visits due to compromised general health. ^12^ However, it is crucial to recognise that this strategy poses a challenge due to the extended intubation hours, which can complicate extubation and postoperative recovery. Fortunately, this concern was effectively addressed in the present case through meticulous time management. In particular, the entire procedure was completed successfully in a time frame of four hours. The primary objective of our multidisciplinary approach was to effectively manage pain, improve oral hygiene, and mitigate degenerative potential.

The collaborative procedure, conducted in coordination with the Department of Otolaryngology and involving adenoidectomy, required oral intubation due to the impracticality of nasal intubation. Unfortunately, this choice compromised the field of vision and accessibility for comprehensive dental rehabilitation. After adenoidectomy, a continuous suction process became imperative to address blood aggregation in the oral cavity and maintain a blood-free field for treatment. Inherent in individuals with Hunter syndrome is the characteristic of macroglossia. After intubation, there was significant tongue enlargement, which exacerbated the challenges related to our field of vision and presented a potential risk of airway obstruction. This situation required vigilant supervision. Additionally, an increased fragility of oral tissues was observed, which further added to the complexity of the procedure. ^13^

The decision to administer general anaesthesia for dental treatment to this child came from the challenging behaviour exhibited on the dental chair. However, the successful completion of full-mouth rehabilitation helped us to focus on implementing preventive strategies. Given the diagnosis of global developmental delay, conventional oral hygiene practices were not feasible, which required the adoption of an innovative and modified preventive regimen. Structural and skeletal deformities associated with these conditions also affect the overall health and well-being of children with Hunter syndrome.

Crucially, ongoing follow-up for such cases is imperative due to reported cases of associated jaw lesions, reminiscent of dentigerous cysts. ^14^^,^^15^ Monitoring, particularly during the second to fourth decades of life, is essential. Recognising these lesions, particularly those associated with unerupted first permanent molars containing pools of chondroitin sulphate B, is significant. Their tendency to worsen with age underscores the need for vigilance and early interventions. The key takeaway is that as paediatric dentists, a comprehensive approach is crucial, particularly for children with syndromes such as Hunter syndrome. Recognising and addressing unique challenges and collaborating across disciplines improve dental care and contribute to overall well-being.

## Consent

Ethical approval was waived for this case report as it does not involve experimental interventions. Patient consent for publication, including written consent for clinical details and images, was obtained from the patient’s father, with confidentiality preserved.

## Data Availability

No data are associated with this article.
