# Supplementary material for: Case Report: Holistic dental care for a child with Hunter syndrome: Addressing dental ramifications, overcoming challenges, and enhancing quality of life
Source: F1000Res. 2024 Apr 15;13:268. [Version 1] doi: 10.12688/f1000research.146468.1 (PMC11134137; doi:10.12688/f1000research.146468.1)
Supplement: Peer review history

# Reviewer report: Reviewer response for version 1

**DOI:** 10.5256/f1000research.160559.r271443

**Published:** 2024-05-28

**Authors:** Raghavendra Shetty

**Affiliations:** 1 Ajman University, Ajman, Ajman, United Arab Emirates

This case report describes the successful management of a child with Hunter syndrome (MPS II) who suffered from severe dental problems and is very well written. The article gives holistic dental care for a child with Hunter syndrome.  It involves the collaborative approach involving multiple departments including the Department of Otolaryngology, which performed endoscopic adenoidectomy, and the Department of Paediatric Surgery, which performed an umbilical and right inguinal herniotomy. The holistic approach to dental care is illustrated very well. 

**Strengths:**

- Describes the importance of dental care for children with Hunter syndrome.
  Provides a detailed account of the challenges associated with dental treatment in such patients, including behavioural issues and underlying physiological factors.
  Emphasizes the benefits of holistic dental care, potentially including full-mouth rehabilitation under general anaesthesia in severe cases.
  Showcases the positive impact of successful treatment on the child's oral health and potentially, their overall quality of life.
**Highlights**
- The report highlights the need for collaboration between pediatric dentists and other healthcare professionals, such as pediatricians, pediatric surgeons and otolaryngologists, to ensure comprehensive care for children with Hunter syndrome.
- Future research could explore effective behaviour management strategies specifically tailored to children with Hunter syndrome undergoing dental treatment.

Overall, this case report offers a valuable contribution by underlining the crucial role of dental care in managing Hunter syndrome and improving the well-being of affected children.

Are enough details provided of any physical examination and diagnostic tests, treatment given and outcomes?

Yes

Is the case presented with sufficient detail to be useful for other practitioners?

Yes

Is sufficient discussion included of the importance of the findings and their relevance to future understanding of disease processes, diagnosis or treatment?

Yes

Is the background of the case’s history and progression described in sufficient detail?

Yes

Reviewer Expertise:

Pediatric dentistry, AI in pediatric dentistry

I confirm that I have read this submission and believe that I have an appropriate level of expertise to confirm that it is of an acceptable scientific standard.

## References

[1] : Evaluation of RURS elbow guard in the management of thumb-sucking habit in children with and without intellectual disability. *Eur Arch Paediatr Dent* .2024;25(1) : 10.1007/s40368-023-00858-3 75-84 10.1007/s40368-023-00858-3 38281253

# Reviewer report: Reviewer response for version 1

**DOI:** 10.5256/f1000research.160559.r271448

**Published:** 2024-05-21

**Authors:** Vivek Padmanabhan

**Affiliations:** 1 RAK Medical and Health Sciences University, Ras Al-Khaimah, United Arab Emirates

The article provides a comprehensive overview of Hunter syndrome, emphasizing its clinical manifestations, diagnostic challenges, and the interdisciplinary approach required for effective management, with a focus on dental care. Here's a constructive review of the article:

 Clarity and Depth of Information: The article effectively communicates complex medical concepts in a clear and understandable manner. It provides detailed information about the clinical manifestations of Hunter syndrome, including both systemic and oral manifestations, which enhances the reader's understanding of the disorder.

 Relevance and Significance: The article highlights the importance of recognizing Hunter syndrome as a rare genetic disorder with significant implications for both systemic health and oral health. It emphasizes the need for genetic counselling and interdisciplinary collaboration in managing the condition, which adds value to the medical literature.

 Case Presentation and Analysis: The case report presented in the article offers valuable insights into the challenges encountered in providing dental care to a child with Hunter syndrome. It effectively illustrates the diagnostic process, treatment interventions, and outcomes, providing practical information for healthcare professionals involved in similar cases.

 Multidisciplinary Approach: The article appropriately emphasizes the importance of a multidisciplinary approach in managing Hunter syndrome, particularly in cases requiring dental rehabilitation under general anaesthesia. It discusses the collaboration between departments such as Pediatrics, Otolaryngology, and Pediatric Surgery, underscoring the holistic care provided to the patient.

 Clinical Insights and Recommendations: The article provides valuable clinical insights into the challenges and considerations specific to dental care in patients with Hunter syndrome. It offers practical recommendations for dental interventions, home care routines, and long-term follow-up, which are useful for healthcare professionals involved in the care of such patients.

 Limitations and Future Directions: While the article provides a comprehensive overview of Hunter syndrome and its management, it could benefit from discussing potential limitations of the presented case report, such as the generalizability of the findings to other patients with Hunter syndrome. Additionally, suggestions for future research directions or areas for further investigation would enhance the article's contribution to the field.

Are enough details provided of any physical examination and diagnostic tests, treatment given and outcomes?

Yes

Is the case presented with sufficient detail to be useful for other practitioners?

Yes

Is sufficient discussion included of the importance of the findings and their relevance to future understanding of disease processes, diagnosis or treatment?

Yes

Is the background of the case’s history and progression described in sufficient detail?

Yes

Reviewer Expertise:

Pediatric Dentistry

I confirm that I have read this submission and believe that I have an appropriate level of expertise to confirm that it is of an acceptable scientific standard.
